# The Foreign Journals

**Published:** 1837-04

**Authors:** 


### Art. XIII.—THE FOREIGN JOURNALS. No. VI.

### GERMAN JOURNALS.

12. *Hannoversche Annalen für die gesammte Heilkunde. Eine Zeitschrift. Herausgegeben von* Dr. G. P. Holscher, Königlichem Leibchirurgus, &c.—*Hanover*, 1836.

*Hanoverian Annals of the Medical Sciences. Edited by* Dr. Holscher, Surgeon to the King of Hanover, &c. Nos. I. II. III. IV., January, April, July, Oct. 1836.

This is another of those new journals which every year sees established in Germany, and which, for the most part, arise to take the place of some others that have closed their brief career. The causes stated by us in the first of this series of notices, as giving rise to such a superfluity of periodical works, necessarily limit the circulation of most of them, and consequently render short the existence of many. As all these journals, particularly on their first appearance, contain valuable communications which can only obtain a small number of readers at best, and which, on the stopping of the publication, are lost in the obscurity which attends unsuccessful undertakings, it is much to be regretted that the originators of periodical works do not lay their plans and calculate their resources with more forethought, care, and caution, so as to ensure a more permanent date, and a more deserved measure of justice for their own labours and those of their friends and supporters. We trust that our brother editor and fellow-subject, Dr. Holscher, has taken care to guard against the evils just mentioned; we can say, with truth, that his new journal, in our opinion, deserves to live; and we cordially wish, both to it and to himself, a long and a prosperous career.

The *Hannoversche Annalen* comes out quarterly, on the same days as our own journal, the four annual numbers constituting a volume of about eight hundred 8vo. pages, price four rix-dollars. It is in the Roman type, and the paper and print are pretty good. It is divided into three departments, *Original Communications*, *Critical Reviews*, and *Miscellanies.* In the numbers before us, we find several valuable memoirs by men of distinguished name in Germany, and some interesting statistical accounts of the medical institutions in the kingdom of Hanover. Among these last, we are much pleased with the general arrangements of a new hospital built within these few years in the city of Hanover, and containing about eighty beds. Judging from the engraved plans and description of this hospital given in the first number, we must say that the whole arrangement strikes us as of a superior kind. We are particularly pleased with the airiness of the whole, and the great number of small apartments for the reception of single cases.

In one article of the Third or Miscellaneous Department, the editor gives a brief summary of medical news, under the title of "Scientific and Bibliographical Intelligence;" and we are flattered by seeing in his second number so favorable a notice taken of the British and Foreign Medical Review and its editors. The appearance of our journal is regarded as a striking proof of the advances made in this country towards a more complete or more just appreciation of the medical science and medical literature of Germany; and we hope we may be allowed to participate in the belief expressed by Dr. Holscher, that our work "will unquestionably and essentially contribute to the extension of the fruits of German industry and German learning in England."

There is a formal notice to contributors in the last page of the first number of this journal, which all editors will allow to be a very sensible one; and we are tempted to transcribe it, in the hope that some other contributors, besides those to the *Hanoverian Annals*, may now and then keep it in mind: it is as follows:

"Our contributors are most respectfully requested—

1. To send their communications in the booksellers' parcels,* (durch Buchändler-Gelegenheit,) to the publishers.

2. To write a second time in the margin, right clerkly, (recht deutlich,) all proper names and technical expressions."

This last request is really important, and, if attended to generally, would save a world of trouble and mistakes. Our German brothers may verily be likened to elephants' trunks: while capable of grasping the greatest things, they disdain not the minutest.

13.—*Wochenschrift für die gesammte Heilkunde. Herausgeber*, Dr. Casper; *Mitredaction*, Dr. Romberg, Dr. V. Stosch, Dr. Thaer.—*Berlin*, 1836.

*Weekly Journal of Medical Science. Editor*, Dr. Casper; *Assistant Editors*, Drs. Romberg, Von Stosch, and Thaer.

This is one of the best of the foreign journals, and, although of small extent, contains probably as great a proportion of valuable materials, in relation to its whole contents, as any periodical medical work now published. This is partly accounted for by the great talents and respectability of the editors, particularly of the chief editor, and by the fact that all the original communications that appear in it *are paid for*, "either at the end of the year, or, if required, immediately after the articles are printed." The *Wochenschrift*, as its name implies, appears weekly, on Saturday, and consists of a single sheet in 8vo., without a cover. It is elegantly printed in the Roman type and on fine white paper, which would be reckoned good even in England. It costs annually three dollars and two-thirds of a dollar, or about ten shillings of our money; i.e. less than twopence halfpenny each number.

The *Wochenschrift* is strictly devoted to medical science, taking no notice of matters of merely temporary interest, whether in the form of medical intelligence or news, medical tittle-tattle or gossip, or medical politics. It is chiefly composed of original communications, but almost always contains one or more very brief critical notices. These seem in general very judiciously and honestly written. In a late number we are pleased to observe justice done to a small English treatise recently translated into German. Well might the reviewer exclaim, "What would the English say if one were to translate into their language a German thing of such *trivialness*?" But this is the character of the Germans: they have such an insatiable appetite for reading, that they are not always nice respecting the quality of the books put before them.

* No doubt by way of saving postage.—Eds.

14.—*Jehrbücher der In- und Ausländischen Gesammten Medecin.* Herausgegeben von C. C. Schmidt, m.d.—*Leipzig.*

*Annals of Medical Science, Domestic and Foreign.* Edited by C. C. Schmidt, m.d. &c.—*Leipzig,* 1834—1837.

Taken as a whole, this is perhaps the most comprehensive, the completest, and largest Journal extant. It commenced in January, 1834, and seems to have been since carried on with great vigour. A striking proof of this, and a feature which is to us quite new, is the fact that it has latterly *anticipated* the proper period of publication by several months. For instance, No. I. of 1837 appeared in November, 1836. This, no doubt, arises from the editors finding, like ourselves, that they have more matter on their hands than their prescribed limits can contain. The Journal is of the largest royal octavo or small folio size, even larger than the Brussels *Encyclographie,* in double columns, each Number consisting of about 140 pages, and three Numbers forming a volume. There are twelve Numbers in the year, and consequently four volumes in each annual series, (*Jahrgang,*) costing twelve Saxon dollars, or about 3*l.* English. It is in the Roman type, and the paper and print are moderately good.

The Journal is divided into six departments, as follows:—Extracts from or condensed notices of the principal papers in all the German and the best of the Foreign Medical Journals, classified under different heads; 2. Clinical Reports, original and selected, of general hospitals, lying-in institutions, and lunatic asylums; 3. Original Communications; 4. Reviews of medical works, German and foreign; 5. Miscellanies, medical Intelligence; 6. Bibliography, or catalogue of medical works published. The matter contained in the first department is most ample and varied, and the critical notices are also numerous and apparently just. The department of original communications is for the most part a blank; and indeed we think this scarcely consistent with the plan of the work. The number of collaborators announced on the cover of this Journal is surprisingly great, being upwards of 220. In the list, indeed, are included all the physicians and surgeons of any note in all the German States, though we cannot believe that one half of them are really active assistants; and indeed, when looking at the names subscribed to the different articles, (and none are anonymous,) this opinion seems justified. It may be safely said, that such a work as this could neither be produced nor supported in any country but Germany; and we can truly add, that it is worthy of the singular industry, curiosity, and enthusiasm of the fine people that inhabit that great country. We formerly recommended Kleinert's Repertorium as giving a complete view of the periodical literature of Germany: we are bound, in justice to Dr. Schmidt's work, to say that it effects this object in a manner equally complete and masterly, and, in addition, notices the periodical literature of other countries, and gives critical reviews of all the medical works of value, wheresoever published, or in whatever type or tongue.

